# Vaccination in the post-COVID era: lessons to be learned in Latin America

**DOI:** 10.3389/fimmu.2025.1726836

**Published:** 2025-12-16

**Authors:** Luis Del Carpio-Orantes, Gabriela Zambrano-Sánchez

**Affiliations:** 1 Department of Internal Medicine, Mexican Social Security Institute, Mexico City, Mexico; 2 Universidad Central de Ecuador Facultad de Ciencias Medicas, Quito, Ecuador

**Keywords:** vaccines, Latin America, long COVID, COVID-19, preventive

## Abstract

In this document we discuss the reality of COVID vaccination in Latin America, which has been uneven across the continent; however, some experiences with COVID vaccination have demonstrated a protective effect against the development of chronic manifestations of COVID, in the so-called post-COVID syndrome.

COVID vaccination has served to reduce morbidity and mortality globally; undoubtedly, some Indian and Arab meta-analyses have shown that vaccination, primarily with mRNA vaccines, has been very effective. Inactivated mRNA-based vaccines and vaccines based on non-replicating viral vectors showed significant protection against the incidence of COVID-19 compared to placebo, with a pooled fold change estimate of 0.08 (95% CI: 0.06-0.10), 0.20 (95% CI: 0.14-0.29), and 0.36 (95% CI: 0.28-0.46), respectively (1).

A German systematic review has highlighted the usefulness of vaccines in the post-COVID condition. COVID-19 vaccines may be moderately effective in preventing this condition. Vaccine effectiveness may increase with the number of doses administered, reaching 70% with three doses of the vaccine (2).

Another meta-analysis conducted in Hong Kong suggests that two-dose vaccination before COVID-19 and one-dose vaccination after COVID-19 are associated with a lower risk of long COVID. Since long COVID significantly reduces quality of life, vaccination could be a possible measure to maintain it, partially protecting against long COVID (3).

In Latin America, vaccination coverage has been uneven. By April 2022, over 70% of the population had received the primary series (two doses), yet access and uptake varied considerably across countries (4). Within this context, PCC remains an underexplored syndrome in the region. The scarcity of studies assessing vaccination as a preventive tool for PCC highlights an important research gap.

Post-COVID-19 conditions (PCC) are estimated to affect 20–30% of unvaccinated individuals within three to six months of SARS-CoV-2 infection (5). This reinforces the critical role of vaccination in reducing the long-term consequences of COVID-19.

Challenges within public health systems, combined with limited awareness among healthcare providers of the wide phenotypic spectrum of PCC, further complicate recognition and diagnosis.

Evidence suggests that SARS-CoV-2 immunization, particularly with multiple doses, reduces the risk of long COVID. A systematic review and meta-analysis by Watanabe et al. found that two vaccine doses lowered the risk of PCC compared with no vaccination (OR = 0.64; 95% CI 0.45–0.92) and provided a significant advantage over a single dose (OR = 0.60; 95% CI 0.43–0.83) (6).

Latin American findings, however, are mixed. Some studies reported no protective effect from one or two doses (57), while others observed a reduction in risk with three or more doses (7, 8). These results contrast with large European population-based studies, where vaccination was strongly associated with a reduced probability of long COVID (6, 9).

The protective effect of additional doses aligns with proposed mechanisms, including reduced viral persistence, improved vascular and endothelial function, stabilization of vagal signaling, and modulation of immune dysregulation (7, 10). Yet neutral results in some Latin American studies suggest that individual and regional factors such as sex, age (11, 12), and comorbidity burden (diabetes, hypertension, dyslipidemia, obesity)—may influence vaccine efficacy.

In Mexican and Brazilian cohorts, PCC patients were typically older, with more comorbidities, and more often unvaccinated individuals who had required supplemental oxygen during acute infection (8, 13–15). While Marra and Núñez reported higher PCC prevalence in men (16, 17), Angarita’s Hispanic cohort found greater odds among women and unvaccinated patients with hypertension or diabetes (14), consistent with other Mexican reports (14). Importantly, this multicenter cohort including Ecuadorian participants (18) described a higher prevalence of sequelae in unvaccinated men or those with only one dose. These patients were more likely to experience respiratory and metabolic complications and to require oxygen support during acute illness.

Across Latin American studies (8), hypertension and diabetes were the most common comorbidities, even among healthcare workers. Notably, one of these studies incorporated genomic sequencing to identify circulating variants, showing that vaccinated individuals were less likely to develop PCC than those infected before vaccination. Reinfection emerged as a strong risk factor, whereas continued immunization appeared protective (19).

The timing of vaccination before or after infection (11), the variant in circulation (Delta vs. Omicron), and vaccine type (12) may all influence outcomes. However, the lack of consistent definitions of PCC complicates the evaluation of severity and duration. Large studies suggest vaccination decreases severe cardiovascular and thromboembolic sequelae of long COVID. Similarly, individuals receiving multiple boosters demonstrated a markedly reduced risk of PCC (20), and some evidence indicates that vaccination with two or more doses may accelerate recovery, reducing symptom persistence (8).

One Mexican hospitalized cohort offered a rare phenotypic characterization of PCC, describing six phenotypes (respiratory, mucocutaneous, neurological, functional, gastrointestinal, and mood/sleep/cognitive disorders) across 23 symptoms, with a median follow-up of 405 days (13). They found no clear reduction in specific phenotypes among vaccinated patients raising the possibility that distinct phenotypes may respond differently to immunization, as suggested in other cohorts (21).

Taken together, the evidence from Latin America suggests that COVID-19 vaccination can reduce both the incidence and duration of PCC, but population characteristics, timing, and vaccination regimens likely shape its effectiveness. The growing regional data highlight PCC as a major public health challenge. There is an urgent need for region-specific research to clarify how vaccination can best prevent these long-term outcomes.

The dismantling of the U.S. Advisory Committee on Immunization Practices (ACIP) has reportedly begun to negatively impact vaccination coverage in the United States, according to the CDC data. Between 2024 and 2025, the percentage of vaccinated children fell to 92.5% for the MMR vaccine (protective against measles, rubella, and mumps) and to 92.1% for diphtheria, tetanus, and pertussis. Although these are still very high immunization levels, they are insufficient to guarantee herd immunity, which requires 95%. All of these actions have a global impact, primarily in unprotected regions such as Latin America and Africa, where there is a struggle to obtain vaccines important for global health (9, 18, 22).

During the COVID-19 pandemic, the politicization of the public health emergency contributed to vaccine hesitancy, not only toward COVID-19 vaccines but also toward other vaccines included in the national immunization programs. Vaccination policies during the COVID-19 pandemic rapidly evolved in response to vaccine mandates, global restrictions, and continuously changing requirements for the population. These policies and shifts generated debates that, in many cases, were not adequately addressed by authorities, leading to confusion and resistance within communities. Restrictions on access to work, public transportation, education, and social activities based on vaccination status contributed to social polarization and further vaccine hesitancy. The resulting decline in trust toward governments and institutions not only impacted COVID-19 vaccination efforts but also undermined well-established routine immunization programs (23, 24).

## Data Availability

The original contributions presented in the study are included in the article/supplementary material. Further inquiries can be directed to the corresponding author.
